# Multipathogen infections in hospitalized children with acute respiratory infections

**DOI:** 10.1186/1743-422X-6-155

**Published:** 2009-09-29

**Authors:** Dan Peng, Dongchi Zhao, Jingtao Liu, Xia Wang, Kun Yang, Hong Xicheng, Yang Li, Fubing Wang

**Affiliations:** 1 Pediatrics Department, Zhongnan Hospital, Wuhan University, Wuhan 430071, PR China; 2 Statistics Department, Public Health Institute, Wuhan University, Wuhan 430071, PR China; 3 Clinical Investigation Department, Zhongnan Hospital, Wuhan University, Wuhan 430071, PR China

## Abstract

**Background:**

To explore the epidemiologic and clinical features of, and interactions among, multipathogen infections in hospitalized children with acute respiratory tract infection (ARTI). A prospective study of children admitted with ARTI was conducted. Peripheral blood samples were analyzed by indirect immunofluorescence to detect respiratory agents including respiratory syncytial virus; adenovirus; influenza virus (Flu) types A and B; parainfluenza virus (PIV) types 1, 2, and 3; chlamydia pneumonia; and mycoplasma pneumonia. A medical history of each child was taken.

**Results:**

Respiratory agents were detected in 164 (51.9%) of 316 children with ARTI. A single agent was identified in 50 (15.8%) children, and multiple agents in 114 (36.1%). Flu A was the most frequently detected agent, followed by Flu B. Coinfection occurred predominantly in August and was more frequent in children between 3 and 6 years of age. A significantly higher proportion of Flu A, Flu B, and PIV 1 was detected in samples with two or more pathogens per sample than in samples with a single pathogen.

**Conclusion:**

Our study suggests that there is a high occurrence of multipathogen infections in children admitted with ARTI and that coinfection is associated with certain pathogens.

## Introduction

Almost two million children die each year from acute respiratory tract infection (ARTI), and most of these children live in developing countries [1]. In developed countries, the incidence of lower respiratory tract infection is high and causes 19% to 27% of hospitalizations in children under the age of 5 years in the USA [2,3]. The etiologic agents of these common infections are respiratory syncytial virus (RSV); adenovirus (Adv); influenza virus (Flu) types A and B; parainfluenza virus (PIV) types 1, 2, and 3; chlamydia pneumonia (CP); and mycoplasma pneumonia (MP) [4].

The relationship between clinical symptoms and respiratory infections has been discussed frequently in the literature, but viral detection provides more specific information on the correlation between clinical symptoms and specific infections [5-7]. With recent advances in methods to detect respiratory agents, numerous studies have shown that some pediatric patients with acute lower respiratory tract infection become infected simultaneously with multiple respiratory viruses [8-10]. However, despite the high rate of infection with viral and other respiratory agents, such as CP and MP, coinfection has received little attention. The aim of this study was to explore the epidemiologic and clinical features of, and interactions among, multipathogen infections in children hospitalized with ARTI in central China.

## Results

### Prevalence of respiratory agents

Respiratory agents were detected in 164 (51.9%) of the 316 children with ARTI. Patients with signs of bacterial infections were excluded from the cohort. A single agent was identified in 50 (15.8%) children and two or more agents in 114 (36.1%). The most frequently detected agent was Flu A (n = 97), followed by Flu B (n = 91), Adv (n = 77), and MP (n = 53). In the 114 specimens with multipathogen infections, the most frequent combinations were Flu A plus Flu B, followed by Adv plus Flu A plus Flu B (Table 1).

**Table 1:** Associations among nine respiratory pathogens in 316 ARTI children

**Pathogens detected**	**Number**	**% of total no. of episodes**	**% of positive episodes**
**Single infection**	50	15.8	30.5
**Coinfection, two pathogens**	52	16.5	31.7
Flu A + Flu B	34	10.8	
Flu-A + Adv	3	0.9	
Adv + PIV 3	3	0.9	
CP + MP	2	0.6	
PIV 1 + PIV 2	2	0.6	
MP + Adv	2	0.6	
Adv + Flu B/RSV	1/1	0.3/0.3	
MP + Flu A/Flu B/RSV/PIV3	1/1/1/1	0.3/0.3/0.3/0.3	
**Coinfection, three pathogens**	37	11.7	22.6
Flu A + Flu B + Adv	20	6.3	
Flu A + Flu B + MP	6	1.9	
Adv + MP + CP	2	0.6	
Flu A + Adv + MP	3	0.9	
Flu B + Adv + MP	1	0.3	
Flu A + MP + CP	1	0.3	
Flu B + PIV 2 + PIV 1	1	0.3	
MP + CP + RSV	1	0.3	
Flu A + Flu B + PIV 2/PIV 1	1/1	0.3/0.3	
**Coinfection, four pathogens**	14	4.4	8.5
Adv + MP + Flu A + Flu B	5	1.6	
Adv + MP + CP + PIV 3	2	0.6	
Adv + Flu A + Flu B + PIV 3/RSV	2/1	0.6/0.3	
Adv + CP + Flu A + Flu B	1	0.3	
Adv + CP + PIV 1 + PIV 2	1	0.3	
Adv + MP + PIV 1 + PIV 3	1	0.3	
MP + PIV 1 + Flu A + Flu B	1	0.3	
**Coinfections, five pathogens**	9	2.8	5.5
Adv + MP + Flu A + Flu B + PIV 3/PIV 1/CP	2/1/1	0.6/0.3/0.3	
Adv + CP + MP + PIV 1 + PIV 2	1	0.3	
Adv + PIV 1 + PIV 2 + PIV 3 + Flu B	1	0.3	
CP + MP + Flu A + Flu B + PIV 3	1	0.3	
Adv + CP + MP + RSV + Flu A/Flu B	1/1	0.3/0.3	
**Coinfections, six pathogens**	2	0.6	1.2
Adv + Flu A + Flu B + PIV 3 + MP + CP	1	0.3	
Adv + Flu A + Flu B + PIV 3 + PIV 1 + PIV 2	1	0.3	
**Total number of coinfections**	114	36.1	69.5
**Total number of episodes with pathogens detected**	164	51.9	
**No pathogen detected**	152	48.1	
**Total number of episodes**	316		

### Age and seasonal distributions

The distribution of agents in the different age groups is presented in Table 2. Flu A infection was detected in 97 patients across all age groups except for the group < 6 months of age. Children between 3 and 6 years of age were infected most frequently by Flu A (53.3%) and Flu B (52.0%). Adv and MP were detected in all age groups although mainly in children between 1 and 6 years of age. PIV infection occurred in 37 children in all age groups. More than 50% of the PIV infections in children between 7 months and 3 years of age were type 3. The highest incidence of infection was in children between 3 and 6 years of age, and one or more agents were detected in 68.0% of the episodes. Coinfection was more frequent in children between 3 and 6 years of age (76.5%) than in other age groups.

**Table 2:** The distribution of agents according to age group

**Agent** **Group**	**1-6 mo; n = 41**		**7 mo-1 yr; n = 53**		**1-3 yr; n = 105**		**3-6 yr; n = 75**		**> 6 yr; n = 42**		**Total; n = 316**	
	**Total no**. **(%)**^a^	**Coinfection** **no. (%)**^b^	**Total no**. **(%)**^a^	**Coinfection** **no. (%)**^b^	**Total no**. **(%)**^a^	**Coinfection** **no. (%)**^b^	**Total no**. **(%)**^a^	**Coinfection** **no. (%)**^b^	**Total no**. **(%)**^a^	**Coinfection** **no. (%)**^b^	**Total no**. **(%)**^a^	**Coinfection** **no. (%)**^b^
RSV	1 (2.4)	1 (100)	3 (5.7)	3 (100)	1 (1.0)	1 (100)	1 (1.3)	1 (100)	0 (-)	0 (-)	6 (1.9)	6 (100)
Adv	10 (24.4)	5 (50)	11 (20.8)	6 (54.5)	28 (26.7)	21 (75.0)	20 (26.7)	18 (90.0)	7 (16.7)	7 (100)	77 (24.4)	58 (75.3)
CP	3 (7.3)	3 (100)	6 (11.3)	6 (100)	4 (3.8)	3 (75.0)	1 (1.3)	1 (100)	3 (7.1)	3 (100)	17 (5.4)	16 (94.1)
MP	6 (14.6)	4 (66.7)	9 (17.0)	8 (88.9)	21 (20.0)	15 (71.4)	12 (16.0)	9 (75.0)	5 (11.9)	4 (80.0)	53 (16.8)	40 (75.5)
Flu A	0 (-)	0 (-)	6 (11.3)	6 (100)	39 (37.1)	36 (92.3)	40 (53.3)	35 (87.5)	12 (28.6)	11 (91.7)	97 (30.7)	88 (90.7)
Flu B	0 (-)	0 (-)	6 (11.3)	6 (100)	33 (31.4)	32 (100)	39 (52.0)	37 (94.8)	13 (31.0)	11 (84.6)	91 (28.8)	86 (94.5)
PIV 1	0 (-)	0 (-)	3 (5.7)	3 (100)	2 (1.9)	2 (97.0)	4 (5.3)	4 (100)	2 (4.8)	2 (100)	11 (3.5)	11 (100)
PIV 2	0 (-)	0 (-)	2 (3.8)	2 (100)	1 (1.0)	1 (100)	3 (4.0)	3 (100)	3 (7.1)	2 (66.7)	9 (2.8)	8 (88.9)
PIV 3	2 (4.9)	2 (100)	5 (9.4)	3 (60)	5 (4.8)	5 (100)	2 (2.7)	2 (100)	3 (7.1)	3 (100)	17 (5.4)	15 (88.2)
Episodes	13 (31.7)	6 (46.1)	22 (41.5)	14 (63.6)	59 (56.2)	31 (52.5)	51 (68.0)	39 (76.5)	19 (45.2)	14 (73.7)	164 (51.9)	114 (69.5)

Note. The age is given at the top of each column.

^a ^Percentage of the total number of episodes investigated in the corresponding age group.

^b ^Percentage of the total number of infections caused by this pathogen in the corresponding age group.

**Figure 1:**
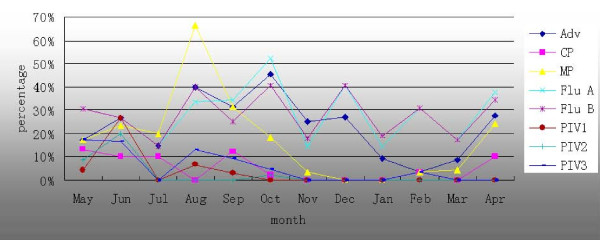
**Monthly distribution of the total percentage of patients**. Adv, adenovirus; Flu, influenza virus; PIV, parainfluenza virus; CP, chlamydia pneumonia; MP, mycoplasma pneumonia.

The monthly distribution of pathogens is shown in Figure 1. The rate of MP infection increased in the early summer and peaked in August. The prevalence of Flu A, Flu B, and Adv peaked in October. CP and PIV 1, 2, and 3 were detected sporadically in a small number of children during the entire study period. Coinfection was more frequent in August (60.0%; 9/15).

### Clinical features

The length of hospital stay and other parameters such as the presence of fever, cough, or vomiting, and white blood cell count did not differ significantly between uninfected children, those with a single infection, or coinfected children (Table 3).

**Table 3:** Clinical presentations in 316 hospitalized ARTI children

**Patient characteristic**	**Negative**^a^ **n = 152**	**Single infection**^a^ **n = 50**	**Coinfection**^a^ **n = 114**
Length of hospital stay (d)	8.44 ± 3.99	7.62 ± 2.91	7.74 ± 2.75
Fever^b^	90	30	83
Vomiting	35	12	25
Cough	113	36	75
Rash	11	2	7
Diarrhea	15	2	5
White blood cells/mm^3^			
< 4000	12	3	12
4000-10000	87	22	62
> 10000	53	24	36
AURI	47	17	40
ALRI	105	33	74

AURI, acute upper respiratory infection; ALRI, acute lower respiratory infection; ^a ^The number in the corresponding group;^b ^Armpit skin temperature ≥ 37.5°C.

### Relationship between the incidence of pathogens and multiple infections

**Figure 2:**
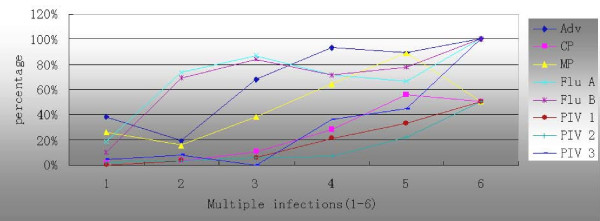
**Correlations between the number of pathogens per sample and the percentage of pathogens**. Adv, adenovirus; Flu, influenza virus; PIV, parainfluenza virus; CP, chlamydia pneumonia; MP, mycoplasma pneumonia.

Figure 2 shows that the incidence of CP, Adv, and PIV 1, 2, and 3 increased with the number of pathogens per sample. The incidence of Flu A and B first increased with the number of pathogens per sample and then decreased when the number of pathogens per sample was more than three. Similarly, the incidence of MP increased with the number of pathogens per sample but decreased when the number of pathogens per sample was six. There was a significantly higher proportion of Flu A (χ^2 ^= 50.398, p < 0.001), Flu B (χ^2 ^= 60.259, p < 0.001), and PIV 1 (χ^2 ^= 5.171, p < 0.05) in samples with two or more pathogens than in those with a single pathogen.

### Binary logistic regression analysis

In the simple logistic regression analysis, infection with Flu B (odds ratio [OR] = 109.71, p < 0.05) or Adv (OR = 3.99, p < 0.05) was an independent factor associated with the incidence of Flu A. In the multivariate logistic regression analysis, only Flu B (OR = 97.22, p < 0.05) was an independent factor (Table 4). In the simple logistic regression analysis, infection with Flu A (OR = 88.98, p < 0.05), Adv (OR = 3.69, p < 0.05), or MP (OR = 1.99, p < 0.05) was an independent factor associated with the incidence of Flu B. In the multivariate logistic regression analysis, only Flu A (OR = 79.504, p < 0.05) was an independent factor. In the simple logistic regression analysis of Adv infection, infection with Flu B (OR = 3.69, p < 0.05), Flu A (OR = 3.72, p < 0.05), CP (OR = 2.97, p < 0.05), MP (OR = 6.47, p < 0.05), PIV 1 (OR = 3.96, p < 0.05), or PIV3 (OR = 11.93, p < 0.05) was an independent factor associated with the incidence of Adv. In the multivariate logistic regression analysis, infection with Flu A (OR = 2.53, p < 0.05), CP (OR = 4.63, p < 0.05), or PIV3 (OR = 11.93, p < 0.05) was an independent factor.

**Table 4:** Cross-correlations of pathogen prevalence rates

**Pathogen-positive** **Background**		**Prevalence (%) of pathogens**						
	
	**Adv**	**CP**	**MP**	**Flu A**	**Flu B**	**PIV 1**	**PIV 2**	**PIV 3**
Adv (77*)		14.3	29.9	**53.2**	50.6	7.8	5.2	16.9
CP (17*)	64.7		**82.4**	35.3	29.4	11.8	11.8	23.5
MP (53*)	43.4	26.4		**45.3**	41.5	7.5	1.9	15.1
Flu A (97*)	42.3	6.2	24.7		**82.5**	4.1	2.1	7.2
Flu B (91*)	42.9	5.5	24.2	**87.9**		6.6	4.4	8.8
PIV 1 (11*)	54.5	18.2	36.4	36.4	54.5		**63.6**	27.3
PIV 2 (9*)	44.4	22.2	11.1	22.2	44.4	**77.8**		22.2
PIV 3 (17*)	**76.5**	23.5	47.1	41.2	47.1	17.6	11.8	

Note. The bold numbers indicate the most cross-correlation of pathogens coinfection. * Number of samples comprising the background-positive group.

For MP, infection with Flu B (OR = 1.995, p < 0.05), Flu A (OR = 2.154, p < 0.05), Adv (OR = 2.967, p < 0.05), CP (OR = 31.111, p < 0.05), or PIV3 (OR = 5.017, p < 0.05) was an independent factor associated with the incidence of MP in the simple logistic regression analysis. In the multivariate logistic regression analysis, only CP (OR = 26.895, p < 0.05) was an independent factor. For CP, infection with PIV 2 (OR = 5.562, p < 0.05), Adv (OR = 6.472, p < 0.05), MP (OR = 31.111, p < 0.05), or PIV3 (OR = 6.769, p < 0.05) was an independent factor associated with the incidence of CP in the simple logistic regression analysis. In the multivariate logistic regression analysis, infection with MP (OR = 41.016, p < 0.05) or PIV 2 (OR = 18.118, p < 0.05) was an independent factor.

For PIV 1, infection with PIV 2 (OR = 265.125, p < 0.05), Adv (OR = 3.955, p < 0.05), or PIV3 (OR = 7.795, p < 0.05) was an independent factor associated with the incidence of PIV 1 in the simple logistic regression analysis. In the multivariate logistic regression analysis, only PIV 2 (OR = 292.808, p < 0.05) was an independent factor. For PIV 2, infection with PIV 1 (OR = 265.125, p < 0.05), CP (OR = 5.562, p < 0.05), or PIV3 (OR = 5.562, p < 0.05) was an independent factor associated with the incidence of PIV 2 in the simple logistic regression analysis. In the multivariate logistic regression analysis, only PIV 1 (OR = 240.106, p < 0.05) was an independent factor. For PIV3, infection with PIV 1 (OR = 7.795, p < 0.05), CP (OR = 6.769, p < 0.05), MP (OR = 5.017, p < 0.05), or PIV 2 (OR = 5.562, p < 0.05) was an independent factor associated with the incidence of PIV3 in the simple logistic regression analysis. In the multivariate logistic regression analysis, only Adv (OR = 9.246, p < 0.05) was an independent factor.

## Discussion

We detected more than one agent in 51.9% of children with a clinical diagnosis of acute respiratory infection. The most frequent combination was coinfection with two agents, primarily Flu A plus Flu B. Six episodes involved coinfection with five agents and two episodes involved coinfection with six agents.

The prevalence of coinfection in previous studies is 11-27% in young children with diverse types of respiratory tract infections seen in the hospital or emergency department [8,11-16], and the most frequent combination was coinfection with two different pathogens. In contrast, we found a much higher incidence of coinfection with more than two agents than that reported previously. The differences in the incidence of coinfection may reflect geographic differences or differences in etiologic agents [17,18] or diagnostic methods [19].

The nine pathogens detected in our study are active in cold and dry environments. It is possible that these agents are associated because they circulate most frequently at the same time of year [15]. In our children, coinfection was observed most frequently between 3 and 6 years of age, probably because of the greater incidence of infection with respiratory pathogens as a whole in this age group [15].

The IIF method to detect antibody to respiratory pathogens may be another reason for the higher rate of coinfection in our study. IIF is a qualitative test to detect antibody that yields initial information about the immune status of the patient, the clinical course of the disease. The presence of antibodies of class IgM does not show whether the child is infected with multiple pathogens at the same time. In most studies, more than 70% of children with an ARTI have detectable levels of IgM antibodies within one week after the onset of infection, and the IgM level declines gradually and becomes undetectable 3 months after the onset of infection. Thus, the IIF method to detect antibodies shows only that a child was infected with a respiratory pathogen more than one week but less than 3 months before the sample was obtained.

In most published studies of dual respiratory viral infections (DRVI), more than one viral diagnostic technique was used to identify respiratory viruses. The rate of DRVI depends on the number of viral diagnostic methods used. When only one diagnostic method was used, the overall rate of DRVI was 1.8%, whereas when two virus detection methods were used, the rate of DRVI was 9.9%, and when three methods were used, the rate was 8.4% [20]. Brunstein et al [21] showed that a direct fluorescence assay backed with a multiplex molecular method is the current best practice in respiratory diagnostics.

The notable findings in our study were the relationship between the incidence of pathogens and multiple infections, and we definitively ruled out preferential interactions among specific agents. A recent study provided statistical evidence that coinfection is not random and that coinfection with certain pathogens occurs more frequently than expected if coinfection was random [21].

Some authors have noted no clinical differences between patients with respiratory infections caused by a single agent or by multiple agents, in hospitalized patients [22] and ambulatory children [23]. In particular, Paranhos-Baccala et al [24] found a significant correlation between DRVI and increased disease severity of bronchiolitis and that dual infection was a risk factor for admission to the pediatric intensive care unit (PICU), independent of the host's condition. However, we found no significant difference in clinical parameters associated with infection by a single pathogen or by multiple agents. All of the five children with CMV infection were coinfected, and most had more than three detectable specific antibodies (IgM) at the same time.

The limitations of our study include the retrospective design and the lack of the use of molecular techniques to detect the respiratory pathogens. Despite these limitations, we identified antibodies to one or more agents in 51.9% of samples.

RSV was detected in only six specimens in this study. RSV is the most common cause of bronchiolitis and pneumonia in infants and young children [25]. A lower percentage of infant enrolment might help explain this finding. We cannot exclude the possibility that the IIF method was not sensitive enough to detect RSV.

In conclusion, we found frequent multipathogen infections in children admitted with ARTIs and a significant relationship between the incidence of pathogens and multiple infection. Further studies are needed to clarify the pathogenesis and interactions involved in coinfection by viruses.

## Materials and methods

### Patients

A total of 316 pediatric patients (≤ 14 years of age) who were hospitalized for respiratory tract infection during the study period May 2008 to April 2009 were investigated in the pediatric department of Zhongnan Hospital of Wuhan University, Hubei province in central China. The respiratory tract infections were classified as upper or lower respiratory tract infection. The symptoms of upper respiratory tract infection (URTI) included cough, sore throat, tonsillitis, pharyngitis, and herpangina. Lower respiratory tract infections (LRTI) included pneumonia, bronchitis, bronchiolitis, and asthma [5]. Infected patients with one or more of these syndromes were included in this study. Children with presumed nosocomial infections (hospital discharge in the previous 2 weeks or onset of respiratory tract infection more than 48 h after admission) were excluded from this cohort. In the same patient, only new episodes occurring at an interval of at least 2 months were included.

A standard medical history was taken from each child and included epidemiological data, clinical antecedents, current disease, clinical manifestations, length of hospital stay, white blood cell count, and chest X-rays taken elsewhere.

### Samples and laboratory methods

A peripheral blood sample was obtained from all children with an ARTI within the first 24 h of admission to the pediatric department. Specific antibodies (IgM) to the infectious agents (RSV; Adv; Flu A and B; PIV 1, 2, and 3; CP; and MP) were detected using a commercial indirect immunofluorescence (IIF) kit (EUROIMMUN, Lübeck, Germany) following the manufacturer's instructions.

### Statistical analysis

General data are presented as the percentage or mean ± SD. All statistical analyses were performed using SPSS software (version 13; Chicago, IL, USA). The chi-square test was used to compare between-group differences in percentages. Binary logistic regression was used to address the factors that might influence the incidence of the pathogens. A p-value < 0.05 was considered significant.

## Abbreviations

IIF: indirect immunofluorescence test; ARTI: acute respiratory tract infection; RSV: respiratory syncytial virus; Adv: adenovirus; Flu: influenza virus; PIV: parainfluenza virus; CP: chlamydia pneumonia; MP: mycoplasma pneumonia.

## Competing interests

The authors declare that they have no competing interests.

## Authors' contributions

PD wrote the manuscript and collected the data; ZD wrote the manuscript and analyzed the data; LJ, WX, YK discussed and reviewed the manuscript; HX and LY provided statistical analysis; and WF detected the blood samples. All authors read and approved the final manuscript.
